# Anaerobic Membrane Bioreactor for Continuous Lactic Acid Fermentation

**DOI:** 10.3390/membranes7020026

**Published:** 2017-05-03

**Authors:** Rong Fan, Mehrdad Ebrahimi, Peter Czermak

**Affiliations:** 1 Institute of Bioprocess Engineering and Membrane Technology, University of Applied Sciences Mittelhessen, Giessen 35390, Germany; rong.fan@lse.thm.de (R.F.); mehrdad.ebrahimi@lse.thm.de (M.E.); 2 Department of Chemical Engineering, Kansas State University, Manhattan, KS 66506, USA; 3 Faculty of Biology and Chemistry, Justus Liebig University Giessen, Giessen 35390, Germany

**Keywords:** anaerobic membrane bioreactor, lactic acid, membrane filtration, membrane fouling, continuous fermentation, optical sensor

## Abstract

Membrane bioreactor systems can enhance anaerobic lactic acid fermentation by reducing product inhibition, thus increasing productivity. In batch fermentations, the bioconversion of glucose is strongly inhibited in the presence of more than 100 g·L^−1^ lactic acid and is only possible when the product is simultaneously removed, which can be achieved by ceramic membrane filtration. The crossflow velocity is a more important determinant of flux than the transmembrane pressure. Therefore, to stabilize the performance of the membrane bioreactor system during continuous fermentation, the crossflow velocity was controlled by varying the biomass concentration, which was monitored in real-time using an optical sensor. Continuous fermentation under these conditions, thus, achieved a stable productivity of ~8 g·L^−1^·h^−1^ and the concentration of lactic acid was maintained at ~40 g·L^−1^ at a dilution rate of 0.2 h^−1^. No residual sugar was detected in the steady state with a feed concentration of 50 g·L^−1^.

## 1. Introduction

Membrane bioreactors (MBRs) are hybrid systems comprising a bioreactor for bioconversion and a membrane device for separation. Dorr-Olivier Inc. reported the first application of crossflow membrane filtration in an activated sludge bioreactor for wastewater treatment [1,2]. In this system, the settling tank in a conventional activated sludge process was replaced with a membrane device, improving the effluent quality, increasing volumetric loading rates and solid retention times, and reducing hydraulic retention times and sludge production [3,4,5,6]. In a further development, membranes were directly immersed in the bioreactor to replace the external separation device [7], significantly reducing the energy costs and footprint. The submerged membranes rely on coarse bubble aeration to minimize fouling, while aeration transfers oxygen to the biomass and maintains the solids in suspension, improving the efficiency of biodegradation. Since these early applications, MBR systems have devolved rapidly to meet diverse requirements in different bioprocesses, reflecting their advantages in terms of biocatalyst immobilization, and simultaneous product generation and recovery. For example, enzyme membrane bioreactors can be used for enzymatic delignification (lignin modification, removal, and utilization) [8,9] or the production of fructo-oligosaccharides [10]. In addition to the aerobic MBR system for wastewater treatment, anaerobic MBRs can be used in fermentation processes to produce organic acids such as itaconic [11], propionic [12], and lactic acids [13,14], because the fermentation of these organic acids generally takes place in anaerobic environments.

Lactic acid (LA) is a versatile carboxylic acid widely used in food products, beverages, cosmetics, and medicines. The demand for LA has increased rapidly due to the production of the biodegradable polymer polylactic acid (PLA), which can be used as a sustainable alternative to petroleum-derived plastics [15,16,17,18,19]. However, the competitiveness of PLA is restricted by the high cost of LA as a raw material. Industrial research has, therefore, focused on strategies to increase the productivity of LA fermentations and reduce the costs. LA is usually produced by the microbial conversion of carbohydrates, such as dextrose, lactose, and sucrose, under anaerobic conditions because this yields a particular stereoisomer rather than the racemic mixture produced by chemical synthesis [20,21,22]. Therefore, we used the Gram-positive, thermophilic, and facultative anaerobic bacterium *Bacillus coagulans* to produce l-LA in the laboratory because this species can achieve high yields under anaerobic fermentation conditions [23,24,25,26].

LA production is dependent on the cell density and growth rate, but the latter is affected by the concentration of LA in the fermentation broth [27,28,29]. In conventional batch or fed-batch processes, the inhibition of cell growth and substrate conversion is unavoidable because LA accumulates in the reactor [30,31]. The MBR system provides a promising solution because membrane filtration allows the continuous removal of LA, while the microorganisms remain in the reactor. Productivity is, therefore, improved by increasing the cell density and reducing product inhibition [13,14,32].

During continuous steady-state fermentation in MBR systems, LA productivity depends on the dilution rate (*D*) as well as the product concentration. The volumetric productivity of a MBR ranges from 8.2 to 33.1 g·L^−1^·h^−1^ and can be controlled by varying either the dilution rate or feed concentration [32,33,34]. Although these approaches may lead to individual side effects because they increase LA productivity via different mechanisms, both the dilution rate and feed concentration can be used to increase the biomass concentration. High cell densities enhance LA productivity but also cause severe membrane fouling, resulting in a rapid decline in the efficiency of the membrane during fermentation therefore necessitating regular membrane cleaning, which increases operational costs [35].

Unlike typical continuous fermentations, the sustainable dilution rate in MBR systems requires a stable flux during filtration, which must be controlled by preventing fouling to ensure the stability of the process. In an anaerobic MBR system, membrane fouling cannot be prevented by aeration, which is the strategy used in aerobic MBR systems for wastewater treatment. The flux must, therefore, be controlled by adjusting the transmembrane pressure (TMP) and/or crossflow velocity (CFV). Furthermore, the physical and biological properties of the feed (such as the components, viscosity, temperature, cell density, and morphology) also influence filtration efficiency [35,36] and must, therefore, be considered during the development of an appropriate control strategy.

Here we aimed to establish an anaerobic MBR system for stable continuous LA fermentation, minimizing product inhibition, and enhancing filtration efficiency by preventing membrane fouling. The complexity of the MBR system during continuous operation requires careful process monitoring and control. Due to the influence of biomass on the stability of the system, we introduced an optical sensor for online monitoring and control, enhancing process optimization for LA production.

## 2. Materials and Methods

### 2.1. Ceramic Membranes

Ceramic membranes with different geometries and pore size/molecular weight cutoff (MWCO) characteristics were used for cell retention and LA recovery. Membrane performance was investigated using tubular ceramic α-Al_2_O_3_ membranes (mono channel) with a nominal MWCO of 100 kDa and an effective area of ~0.0043 m^2^ (atech innovations GmbH, Gladbeck, Germany). UF membranes with the equivalent pore size (35 nm) were measured by the manufacturer using the mercury pressure porosimetry method. In order to ensure the desired flux for continuous fermentation, a hollow fiber membrane with a larger effective area (~0.033 m^2^) was used to establish the MBR system. The 31-fiber ceramic hollow fiber membrane (Mann + Hummel GmbH, Ludwigsburg, Germany) had a nominal pore size of 40 nm.

### 2.2. Bacterial Culture

*Bacillus coagulans* PS5, a facultative anaerobic homofermentative l(+) LA-producing bacterium, was kindly supplied by thyssenkrupp Industrial Solutions AG, Process Technologies (Leuna, Germany). The culture medium was modified MRS (de Man, Rogosa, and Sharpe) medium, which contained 4.0 g·L^−1^ yeast extract, 8.0 g·L^−1^ meat extract, 10.0 g·L^−1^ peptone from casein, 2 g·L^−1^ K_2_HPO_4_, 0.1 g·L^−1^ MgSO_4_, 0.03 g·L^−1^ MnSO_4_, 1.0 g·L^−1^ Tween-80, and 20 g·L^−1^ glucose. The inoculum was grown in the same medium. All components were sterilized at 121 °C for 20 min except the glucose, which was sterilized separately.

### 2.3. Batch Fermentation

The batch fermentations were carried out in a 5-L Biostat^®^ B stirred bioreactor (Sartorius Stedim Biotech GmbH, Göttingen, Germany) at 53 °C with neutralization achieved by adding 5 M NaOH. The influences of fermentation conditions, e.g., glucose concentration and pH value, on the cell growth and LA production were determined to investigate the fermentation kinetics, as well as the optimum culture condition.

### 2.4. Optical Sensor

An EXcell 230 optical sensor, kindly provided by Exner Process Equipment GmbH (Ettlingen, Germany), was used to measure the cell density during fermentation. The EXcell 230 is an online monitoring device based on 880 nm near infrared (NIR) absorbance measurements, which can be used to measure OD values in microbial fermentations. The optical path length of the sensor is 10 mm, which can measure cell densities of up to 200 g·L^−1^. The original measurement result was displayed in arbitrary units (AU).

### 2.5. Experimental Equipment

**Figure 1:**
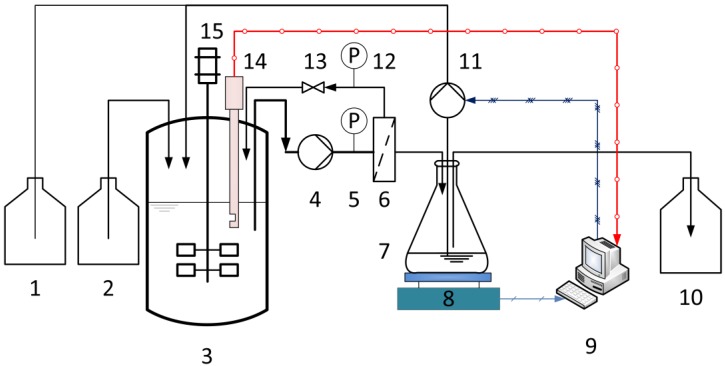
Membrane bioreactor system with online biomass monitoring using the optical sensor for continuous LA fermentation: (1) base, (2) culture medium, (3) stirred tank reactor, (4) rotary vane pump, (5,12) manometers, (6) membrane module, (7) intermediate reservoir, (8) electric balance, (9) control system, (10) product, (11) peristaltic pump, (13) valve, (14) optical sensor, and (15) motor.

The configuration of the experimental equipment for membrane characterization and continuous fermentation is shown schematically in Figure 1. In order to increase the dilution rate (*D*), the fermentations were carried out in a 1.5-L Biostat^®^ B stirred bioreactor (Sartorius Stedim Biotech GmbH, Göttingen, Germany) instead of the 5-L bioreactor. The pH value was maintained at 6.4 by adding 10 M NaOH. The optical sensor was plugged directly into the bioreactor. The fermentation broth was pumped through the membrane module using a rotary vane pump. The system was set to one of two operational configurations. In the first configuration, when no fresh medium was fed, the membrane was characterized at different TMPs and CFVs using a crossflow filtration system in total recycle mode. Here, the permeate and retentate were returned to the fermenter in order to maintain the concentration and temperature of the fermentation broth. Second, when the continuous fermentation started, the fresh medium was fed into the reactor with a multichannel peristaltic pump. In the meantime, the permeate was first collected in an intermediate reservoir, the weight being monitored using an electric balance. During the continuous operation, the permeate flux must be maintained above a minimum level (desired flux), which is determined by the set dilution rate. Therefore, the collected permeate was partly removed as the product with the peristaltic pump. The output flow was set equal to the input flow (fresh medium) so that the operating volume remained constant, and the rest permeate was then recycled back to the reactor, once the volume of permeate in the intermediate reservoir exceeded 100 mL.

The performance of the MBR system in continuous fermentations was determined by the dilution rate and the feed concentration. *D* was calculated according to Equation (1), and controlled by the varying the TMP and CFV.

(1) $$D=\frac{F_{in/out}}{V}$$

where *F_in/out_* refers to the volumetric flow rate of the medium/product, and *V* is the operating volume of the reactor.

The TMP and CFV were controlled for the desired flux using the rotary vane pump and a stainless steel valve, mounted on the retentate outlet. Permeate flow was monitored online with a miniature oval gear flow meter (B.I.O-Tech e.K., Vilshofen, Germany). The membrane flux was calculated according to Equation (2) once the experiment was completed:

(2) $$J=\frac{F}{A}$$

where F refers to the volumetric flow rate of the permeate, and *A* is the membrane effective area.

The TMP was varied between 0.2 and 2.5 bar, and the CFV was varied between 0.5 and 3.0 m·s^−1^. Since the permeate side was open during the experiments, the pressure on the permeate side was equal to atmospheric pressure. Therefore, the TMP was measured and monitored using two manometers located on the inlet (*P*_1_) and outlet (*P*_2_) of the membrane module, and was calculated using Equation (3):

(3) $$\mathrm{TMP}=\frac{P_{1}+P_{2}}{2}$$

The CFV (m·s^−1^) was calculated using the quotient of the retentate volumetric flow rate per inner cross-sectional area of the membrane tube.

After each filtration run, the membranes were rinsed with 2–3 L tap water and 2–3 L RO water, respectively. The membranes were then rinsed with 2 L alkaline 1% Asiral^®^ (Asiral Industrie-Reiniger GmbH, Neustadt, Germany) at 53 °C for 1 h to remove the membrane fouling.

### 2.6. Analytical Method

Glucose and lactate concentrations were measured using Biosen C line GP (EKF-diagnostic GmbH, Barleben, Germany). The OD was measured using a UV-VIS spectrophotometer (Eppendorf AG, Hamburg, Germany). An aliquot was diluted with reverse osmosis (RO) water to ensure the absorbance was <0.5. The OD of the dilution was then measured three times at 600 nm (*OD*_600_). To determine the CDW, 10-mL aliquots of cells were collected by centrifugation (10,000 rcf, 10 min, 4 °C) and transferred to a dried, pre-weighed 2-mL centrifuge tube. The cells were then resuspended in RO water and centrifuged at 16,100 rcf for 2 min at 4 °C (Eppendorf AG). The pellets were washed, centrifuged twice as above and dried at 55 °C until a constant mass was obtained.

### 2.7. Calculation

The product-substrate yield (*Y_p/s_*) was defined as the increase in LA per consumed glucose (Equation (4)):

(4) $$Y_{p/s}=\frac{∆c_{LA}}{∆c_{glu}}$$

The productivity of LA ($Q_{p}$) was defined as the increase in LA per unit of time during fermentation (Equation (5)). The overall productivity of LA during the batch fermentation (${\bar{Q}}_{p}$) was then equal to the final LA concentration divided by the entire fermentation time. For continuous fermentation, the productivity of LA was calculated with the concentration of LA in the outlet flow and the dilution rate according to Equation (6):

(5) $$Q_{p}=\frac{∆c_{LA}}{∆t}$$

(6) $$Q_{p}=c_{LA}\times D$$

The specific growth rate (μ) was measured using the EXcell sensor, *OD*_600_ values and CDW, and was used to characterize the kinetics of the LA fermentation. The data obtained from the optical sensor were fitted to Equation (7) to calculate the maximum specific growth rate (μ_max_), where *N* refers to data captured during the exponential phase:

(7) $$N=N_{0}\times e^{µ\times t}$$

For offline measurement, μ was defined as the increase in biomass per unit time (Equation (8)), where *M* is the *OD*_600_ value or CDW, as appropriate:

(8) $$µ=\frac{\ln\frac{M_{t2}}{M_{t1}}}{t_{2}-t_{1}}$$

## 3. Results

### 3.1. Batch Fermentation for LA Production

**Figure 2:**
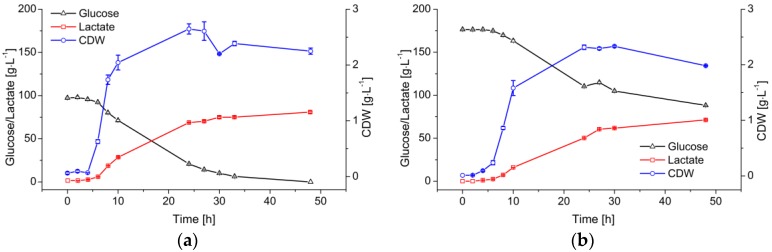
Profile of LA production from glucose by *B. coagulans* in a 5-L fermenter containing 3 L medium, at pH 6.0 and 53 °C. (**a**) Initial glucose = 100 g·L^−1^; (**b**) Initial glucose = 180 g·L^−1^. Values are means ± standard deviation (*n* = 3).

Figure 2 compares fermentations using *B. coagulans* with two initial glucose feed concentrations (100 and 180 g·L^−1^). Both fermentations started with 1/30 (*v*/*v*) inoculum. The cells reached the exponential phase after a 4-h lag phase. The μ_max_ values achieved with the two initial glucose concentrations were 1.1 ± 0.1 and 0.9 ± 0.1 h^−1^, respectively. Although the cell growth gradually declined after 10 h, the cell dry weight (CDW) kept increasing to a maximum at ~25 h.

The decreasing cell growth rate also caused a gradual decline in the glucose consumption and LA production rates. The 100 g·L^−1^ initial glucose was completely consumed during the first 48 h of the fermentation, and was converted into LA with a product-substrate yield (*Y_p/s_*) of 0.84 g·g^−1^. The overall LA productivity (${\bar{Q}}_{p}$) was 1.61 ± 0.12 g·L^−1^·h^−1^. When the initial glucose increased to 180 g·L^−1^, the final concentration of LA did not increase significantly because there was ~90 g·L^−1^ of residual sugar.

### 3.2. Filtration Performance of Ceramic Membranes

**Figure 3:**
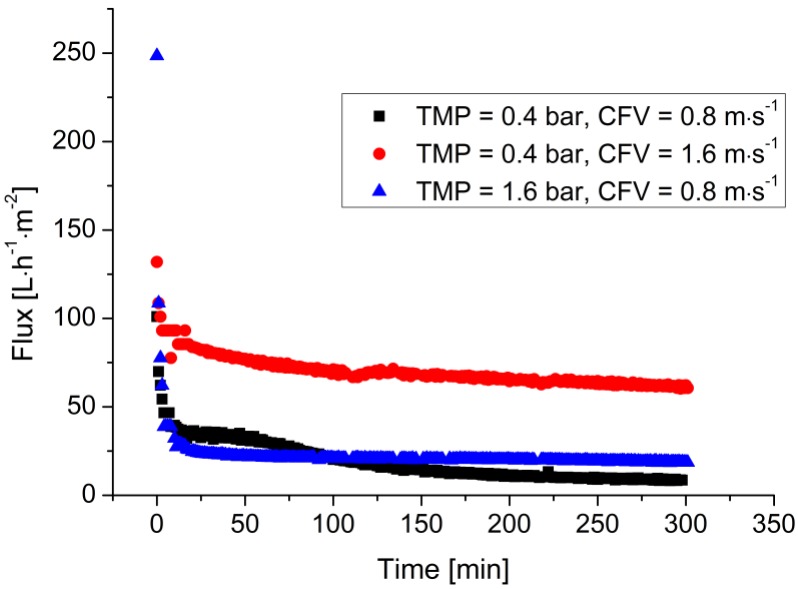
Filtration of the LA fermentation broth under different operating conditions. Cell density = ~2.5 g·L^−1^, LA concentration = ~45 g·L^−1^, *T* = 53 °C, 100 kDa ceramic membrane.

Figure 3 shows the filtration of the LA fermentation broth using a 100 kDa membrane under different operating conditions. The permeate flux strongly decreased within 30 min and gradually reached the steady state. When the CFV increased from 0.8 to 1.6 m·s^−1^, the flux at 6 h increased from 10 to 75 L·m^−2^·h^−1^. In contrast, the flux only increased to 20 L·m^−2^·h^−1^ when the TMP was increased to 1.6 bar at a constant CFV of 0.8 m·s^−1^.

### 3.3. Dynamic Calibration of the EXcell Sensor

**Figure 4:**
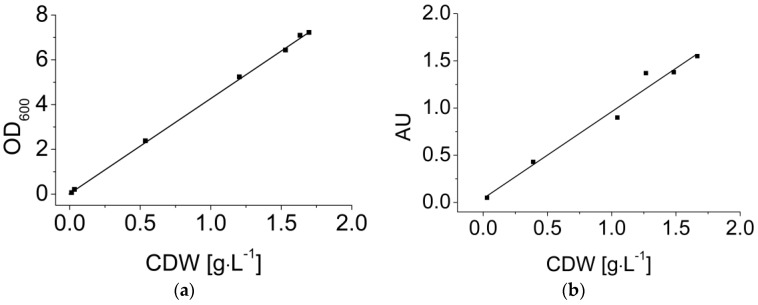
Dynamic calibration of (**a**) optical density (*OD*_600_) and (**b**) the EXcell sensor in *B. coagulans* LA fermentation broth. CDW: cell dry weight. The sensor values in each sample were recorded every 10 s for a total duration of 1 min. All data are shown in the figure.

Dynamic calibration is necessary because optical measurements during the fermentation depend on the varying biomass concentration. Offline measurements, i.e., the OD measurement and direct weighing, were used as reference methods. As an example, Figure 4 shows dynamic calibrations of the OD measurement (a) and the EXcell sensor (b) with fermentation broth containing *B. coagulans*. Both of the OD measurements and the sensor values showed a linear correlation with the biomass concentration up to 2 g·L^−1^ (Equations (9) and (10)).

(9) $${\mathrm{OD}}_{600}=4.3\times \mathrm{CDW}\left(g\right)\;\mathrm{with}\;R^{2}=0.999$$

(10) $$\mathrm{AU}=0.92\times \mathrm{CDW}\left(g\right)+0.04\;\mathrm{with}\;R^{2}=0.978$$

### 3.4. Continuous Fermentation of LA in the MBR

**Figure 5:**
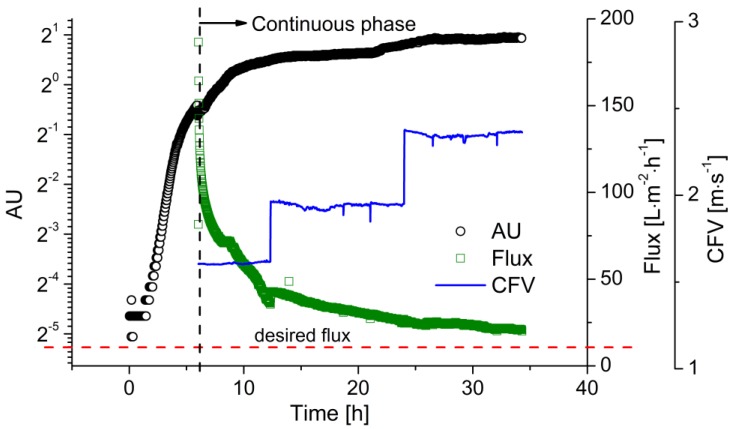
The filtration flux during continuous LA fermentation with flux control. The biomass concentration was monitored using the EXcell optical sensor. Membrane: ceramic hollow fiber membrane, *T* = 53 °C, pH = 6.4, *D* = 0.2 h^−1^, *c_glu_* = 50 g·L^−1^, TMP = 0.4 bar, CFV = 1.6–2.4 m·s^−1^.

The higher productivity of the continuous process requires a continuous supply of substrate. Therefore, before the start of continuous fermentation, a batch fermentation phase is necessary to accumulate enough viable cells in the reactor with a sufficient growth rate to convert the fed substrate. Once cell growth reaches the deceleration phase, as indicated by the optical sensor, the process can be switched to continuous operation. Figure 5 shows the time course of biomass and flux during a continuous fermentation as an example of two replications under this condition. The batch fermentation was carried out with *c_glu_* = 20 g·L^−1^ at pH 6.4. Two hours after inoculation, cell growth reached the exponential phase, which is clearly represented by the straight line in the semi-logarithmic growth plot. As the glucose was gradually consumed, cell growth slowed down due to substrate limitation. Before the glucose was exhausted, fresh medium with a glucose concentration of 50 g·L^−1^ was fed into the reactor. The process was then switched to continuous operating mode. In comparison to the growth curve in the batch processes, the cell density kept increasing throughout the process, rather than immediately declining after the consumption of the substrate. Meanwhile, the flux strongly declined during the fermentation, falling from 180 to 30 L·m^−2^·h^−1^ due to membrane fouling within the first 6 h of filtration. When the sensor readout reached ~1.50, the CFV was increased from 1.6 to 2.0 m·s^−1^. The flux then increased to 40 L·m^−2^·h^−1^. Over the following 18 h, the flux declined to 25 L·m^−2^·h^−1^, while the sensor readout increased to ~1.80. The CFV was then increased to 2.4 m·s^−1^, preventing further flux decline, successfully maintaining the flux above the critical level, and ensuring the stability of the entire continuous process.

**Figure 6:**
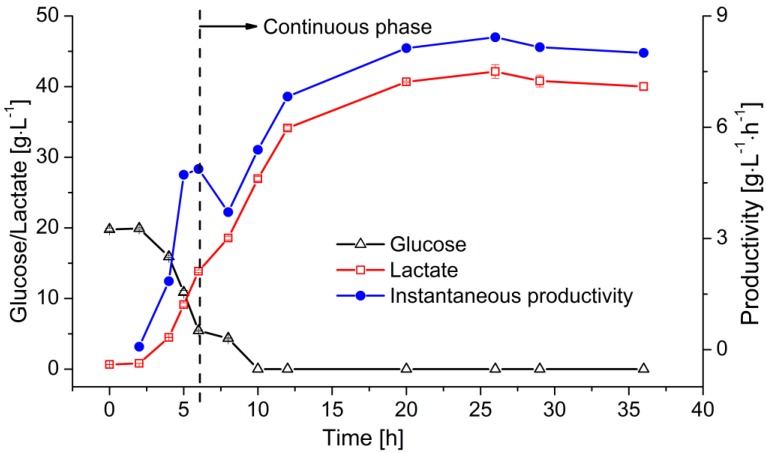
Continuous LA fermentation in a MBR system. Membrane: ceramic hollow fiber membrane concentration values (*T* = 53 °C, pH = 6.4, *D* = 0.2 h^−1^, *c_glu_* = 50 g·L^−1^) are means ± standard deviation (*n* = 3).

At the end of the batch operation, the instantaneous LA productivity reached 5–6 g L^−1^ h^−1^. The permeate containing LA was subsequently removed from the reactor by filtration and fresh medium was added to supply the required substrate. Figure 6 shows the change in glucose and lactate concentration, as well as the instantaneous productivity of the reactor. The residual sugar was consumed within 6 h. The remaining glucose was then converted into LA leaving no residual sugar. However, the lactate concentration kept increasing within the first 20 h of the continuous fermentation due to the continuous glucose supply. After reaching its maximum of 42 g·L^−1^, the lactate concentration stabilized at ~40 g·L^−1^ with the product-substrate yield of 0.8 g·g^−1^. The productivity of this system reached ~8 g·L^−1^·h^−1^ at the steady state.

## 4. Discussion

### 4.1. Batch Fermentation

Studies of LA production have mostly focused on increasing the final concentration of LA in the fermentation broth [37,38,39] because a high titer reduces downstream processing costs during industrial production [31]. In batch fermentations, higher LA titers are usually achieved by increasing the initial substrate concentration. However, due to product inhibition, the consumption of glucose and the production of LA gradually declines as LA accumulates in the reactor [26,39]. When the concentration of LA exceeds a certain threshold, the residual sugar cannot be utilized and is wasted. Furthermore, increasing the substrate concentration does not usually increase the productivity to more than 6 g·L^−1^·h^−1^ [15,25,26,37,38,39] because the duration of the entire fermentation is prolonged. Therefore, in situ product removal, e.g., by membrane filtration, is a promising approach to improve productivity by preventing product inhibition.

### 4.2. Filtration of the Fermentation Broth

According to the mass transfer model of membrane filtration, the flux is proportional to TMP and is not affected by the CFV, but only when only pure water is filtered [35,36,40]. The situation becomes more complex when the fermentation broth is filtered because the components can foul the membrane via mechanisms, such as concentration polarization and cake layer formation [36,40,41]. Our previous investigation indicated that the cake layer formed by cells deposited on the membrane surface is the major cause of resistance during the filtration of *B. coagulans* fermentation broth [35]. Due to the high length/diameter ratio of *B. coagulans*, the cake layer is sensitive to changes in pressure. At the low pressure of 0.4 bar, the loose cake layer can be removed easily by applying a high CFV. In contrast, the cake layer is strongly compressed at high TMP, causing a much greater resistance. Therefore, increasing the CFV may improve the flux more effectively than increasing the TMP.

### 4.3. Dynamic Calibration of the Optical Sensor

Calibration is generally necessary for the quantitative measurement of an objective using an optical sensor because the sensor signal varies in different particle systems due to differences in particle morphology. The excellent linear correlation with the OD measurement suggests that the cell morphology did not vary significantly during the fermentation. Our results demonstrate that the OD values can accurately represent the increase in biomass concentration. Meanwhile, the values reported by the EXcell sensor showed a linear correlation with the biomass concentration. EXcell sensor values could, therefore, be used to replace offline measurements for quantitative calculations such as the determination of μ_max_, because the cell density at the end of exponential phase (~0.5 g·L^−1^) did not extend beyond the linear range of the sensor signal. The EXcell sensor is more convenient for quantitative measurements than some other types of optical sensors, such as scattering light sensors, which often require complicated non-linear regression [42]. Additionally, the sensor value continued to show a positive correlation when the cell density was beyond the linear range. Therefore, the EXcell sensor was used in our MBR system for the online monitoring of cell growth.

### 4.4. Continuous Fermentation of LA in the MBR

The MBR system has always suffered from severe membrane fouling, in particular when the cell number increases continuously [43]. The stable operation of a continuous MBR fermentation is, therefore, difficult to achieve in the absence of fouling control [44]. Considering the high compressibility of the cake layer, previous investigations suggested that a lower TMP may be preferable for filtration during continuous fermentation [35,44]. Therefore, we carried out the continuous fermentation at 0.4 bar, and applied stepwise increases in the CFV to minimize fouling, thus maintaining the flux above the desired level. Online monitoring using the optical sensor indicated the most appropriate time points to increase the CFV, enhancing the convenience and reliability of process control.

MBRs not only aim to improve the stability of continuous fermentation but also the efficiency of LA production, i.e., they aim to achieve a high titer of LA and sufficient substrate utilization. High titers of LA in the permeate are generally achieved by ensuring a high feed concentration. However, this often causes a buildup of residual sugar in the permeate [32,45]. In our previous investigation, the residual sugar concentration rose to a maximum of 20 g·L^−1^ when 70 g·L^−1^ glucose was fed at a dilution rate of 0.2 h^−1^ [44]. Here, the same LA concentration was achieved with a lower feed concentration of 50 g·L^−1^ and no residual sugar was detected, greatly minimizing substrate waste.

The performance of an MBR system, e.g., productivity of LA, consumption rate of substrate, product yield, can strongly influence the production cost of LA. Table 1 compares the general performance of our system with the other fermentation systems reported in the literature:

In addition to the operation mode of fermentation, various previous investigations in the literature also used approaches from different aspects to intensify the LA production, reducing its production cost, e.g., utilizing economical feedstock sources [50,51,52], selecting highly-productive strains of microorganisms [53], and optimizing fermentation conditions [31,54]. Moreover, the lactate in the permeate can be treated further by other membrane processes, e.g., bipolar electrodialysis can convert it into LA, while regenerated alkaline can be reused for the neutralization of the fermentation broth [55,56,57]. Subsequently, the generated LA can be purified and concentrated by nanofiltration, further improving the process efficiency [32].

## 5. Conclusions

We successfully established an anaerobic MBR system for continuous LA fermentation and demonstrated that it can increase productivity mainly because cell retention achieved by membrane filtration increases the cell density, and continuous product removal alleviates the inhibitory effect of accumulated LA. This investigation provided an alternative approach to enhance the efficiency of fermentations that currently suffer from product inhibition. The fouling control strategy, based on stepwise increases in CFV, ensures the desired flux is maintained during continuous fermentation, increasing the operating life of the membrane, and reducing the operating cost. The use of an optical sensor for the online monitoring of cell density provided more insights into this process. The productivity of the MBR system in terms of LA yield was five times higher than in conventional batch processes.

## Figures and Tables

**Table 1:** Overview of different fermentation systems for LA production.

Operation Mode	MO ^1^	Substrate Conc. ^2^ (g·L^−1^)	LA Conc. (g·L^−1^)	Dilution Rate (h^−1^)	Yield (g·g^−1^)	Productivity (g·L^−1^·h^−1^)	Ref.
Continuous with total cell recycle	*B. coagulans* PS5	50	42	0.2	0.84	8.4	This work
Batch	*B. coagulans* WCP 10-4	240	210	–	0.95	3.5	[31]
Fed-batch	*B. coagulans* C106	120 + 80 + 60	215.7	–	0.95	4.0	[39]
Membrane integrated repeated batch	*B. coagulans* IPE22	60	56.5	–	0.96	2.35	[46]
Membrane integrated repeated batch	*B. coagulans* NBRC 12714	110	91.4	–	0.90	3.9	[38]
Continuous with cell recycle	*B. coagulans* NBRC 12714	100	92	0.15	0.92	13.8	[38]
Continuous with cell recycle	*B. coagulans* AD	51	35.2	0.167	0.95	3.69	[47]
Continuous with cell recycle in two-stages MBR	*S. bovis*	30	20.1	0.25	0.67	5.03	[48]
Continuous in MBR with cell bleeding	*B. coagulans* ATCC 23498	99.7	85.4	0.357	0.86	30.5	[49]

^1^ MO: microorganism. ^2^ Substrate concentration refers to the initial total sugar concentration for batch fermentation, or total feed concentration for continuous fermentation.

## Abbreviations

The following abbreviations are used in this manuscript:

AU	Arbitrary units
CDW	Cell dry weight
CFV	Crossflow velocity
*F*	Flow rate
LA	Lactic acid
μ	Specific growth rate
*M*	Mass of biomass represented by offline measurements
MBR	Membrane bioreactor
MWCO	Molecular weight cutoff
MO	Microorganism
*N*	Mass of biomass represented by online measurements
NIR	Near infrared
*OD_x_*	Optical density at *λ* = *x* nm
*P*	Pressure
PLA	Polylactic acid
*Q*	Productivity of lactic acid
RO	Reverse osmosis
*t*	Time
*T*	Temperature
TMP	Transmembrane pressure

## References

[1] Le-Clech P., Chen V., Fane T.A. (2006). Fouling in membrane bioreactors used in wastewater treatment. J. Membr. Sci..

[2] Smith C.V., Di Gregorio D., Talcott R.M. The use of ultrafiltration membranes for activated sludge separation. Proceedings of the 24th Annual Purdue Industrial Waste Conference.

[3] Iorhemen O.T., Hamza R.A., Tay J.H. (2016). Membrane Bioreactor (MBR) Technology for Wastewater Treatment and Reclamation: Membrane Fouling. Membranes.

[4] Meng F., Chae S.-R., Drews A., Kraume M., Shin H.-S., Yang F. (2009). Recent advances in membrane bioreactors (MBRs): Membrane fouling and membrane material. Water Res..

[5] Mutamim N.S.A., Noor Z.Z., Hassan M.A.A., Yuniarto A., Olsson G. (2013). Membrane bioreactor: Applications and limitations in treating high strength industrial wastewater. Chem. Eng. J..

[6] Judd S. (2008). The status of membrane bioreactor technology. Trends Biotechnol..

[7] Yamamoto K., Hiasa M., Mahmood T., Matsuo T. (1989). Direct Solid-Liquid Separation Using Hollow Fiber Membrane in an Activated Sludge Aeration Tank. Water Sci. Technol..

[8] Busse N., Kraume M., Czermak P. (2016). Optimal permeate flux for an enzymatic oxidation of technical lignins in a membrane reactor. Sep. Sci. Technol..

[9] Ebrahimi M., Busse N., Kerker S., Schmitz O., Hilpert M., Czermak P. (2016). Treatment of the Bleaching Effluent from Sulfite Pulp Production by Ceramic Membrane Filtration. Membranes.

[10] Ur Rehman A., Kovacs Z., Quitmann H., Ebrahimi M., Czermak P. (2016). Enzymatic production of fructo-oligosaccharides from inexpensive and abundant substrates using a membrane reactor system. Sep. Sci. Technol..

[11] Carstensen F., Klement T., Büchs J., Melin T., Wessling M. (2013). Continuous production and recovery of itaconic acid in a membrane bioreactor. Bioresour. Technol..

[12] Colomban A., Roger L., Boyaval P. (1993). Production of propionic acid from whey permeate by sequential fermentation, ultrafiltration, and cell recycling. Biotechnol. Bioeng..

[13] Pal P., Dey P. (2013). Process intensification in lactic acid production by three stage membrane integrated hybrid reactor system. Chem. Eng. Process..

[14] Lu Z., Wei M., Yu L. (2012). Enhancement of pilot scale production of l(+)-lactic acid by fermentation coupled with separation using membrane bioreactor. Process. Biochem..

[15] Subramanian M.R., Talluri S., Christopher L.P. (2015). Production of lactic acid using a new homofermentative Enterococcus faecalis isolate. Microb. Biotechnol..

[16] Wee Y.-J., Kim J.-N., Ryu H.-W. (2006). Biotechnological Production of Lactic Acid and Its Recent Applications. Food Technol. Biotechnol..

[17] Rosenberg M., Rebroš M., Krištofíková L., Malátová K. (2005). High Temperature Lactic Acid Production by *Bacillus coagulans* Immobilized in LentiKats. Biotechnol. Lett..

[18] Castillo Martinez F.A., Balciunas E.M., Salgado J.M., Domínguez González J.M., Converti A., de Souza Oliveira R.P. (2013). Lactic acid properties, applications and production: A review. Trends Food Sci. Technol..

[19] Quitmann H., Fan R., Czermak P. (2014). Acidic organic compounds in beverage, food and feed production. Adv. Biochem. Eng. Biotechnol..

[20] Chahal S.P., Starr J.N. (2006). Lactic Acid, Ullmann’s Encyclopedia of Industrial Chemistry.

[21] Narayanan N., Roychoudhury P.K., Srivastava A. (2004). l(+) lactic acid fermentation and its product polymerization. Electron. J. Biotechnol..

[22] Zhao B., Wang L., Li F., Hua D., Ma C., Ma Y., Xu P. (2010). Kinetics of d-lactic acid production by *Sporolactobacillus sp.* strain CASD using repeated batch fermentation. Bioresour. Technol..

[23] De Vecchi E., Drago L. (2006). *Lactobacillus sporogenes* or *Bacillus coagulans:* Misidentification or Mislabelling?. Int. J. Probiotics Prebiotics.

[24] Xu P., Wang L., Zhao B., Ma C., Su F., Tao F., Tang H. (2013). *Bacillus coagulans* Strains and their Applications in l-Lactic Acid Production. U.S. Patent.

[25] Michelson T., Kask K., Jõgi E., Talpsep E., Suitso I., Nurk A. (2006). l(+)-Lactic acid producer *Bacillus coagulans* SIM-7 DSM 14043 and its comparison with *Lactobacillus delbrueckii* ssp. lactis DSM 20073. Enzyme Microb. Technol..

[26] Walton S.L., Bischoff K.M., Heiningen A.R.P., Walsum G.P. (2010). Production of lactic acid from hemicellulose extracts by *Bacillus coagulans* MXL-9. J. Ind. Microbiol. Biotechnol..

[27] Luedeking R., Piret E.L. (1959). A kinetic study of the lactic acid fermentation. Batch process at controlled pH. Biotechnol. Bioeng..

[28] Zacharof M.-P., Lovitt R.W. (2013). Modelling and simulation of cell growth dynamics, substrate consumption, and lactic acid production kinetics of *Lactococcus lactis*. Biotechnol. Bioprocess. Eng..

[29] Nandasana A.D., Kumar S. (2008). Kinetic modeling of lactic acid production from molasses using *Enterococcus faecalis* RKY1. Biochem. Eng. J..

[30] Ou M.S., Ingram L.O., Shanmugam K.T. (2011). l(+)-Lactic acid production from non-food carbohydrates by thermotolerant *Bacillus coagulans*. J. Ind. Microbiol. Biotechnol..

[31] Zhou X., Ye L., Wu J.C. (2013). Efficient production of l-lactic acid by newly isolated thermophilic *Bacillus coagulans* WCP10-4 with high glucose tolerance. Appl. Microbiol. Biotechnol..

[32] Dey P., Pal P. (2012). Direct production of l(+) lactic acid in a continuous and fully membrane-integrated hybrid reactor system under non-neutralizing conditions. J. Membr. Sci..

[33] Xu G.-Q., Chu J., Wang Y.-H., Zhuang Y.-P., Zhang S.-L., Peng H.-Q. (2006). Development of a continuous cell-recycle fermentation system for production of lactic acid by *Lactobacillus paracasei*. Process. Biochem..

[34] Cirilo N.H., Toshiyuki M., Genta K., Kenji S., Ayaaki I. (2002). Synchronized fresh cell bioreactor system for continuous l-(+)-lactic acid production using *Lactococcus lactis* IO-1 in hydrolysed sago starch. J. Biosci. Bioeng..

[35] Fan R., Ebrahimi M., Quitmann H., Czermak P. (2015). Lactic acid production in a membrane bioreactor system with thermophilic *Bacillus coagulans*: Fouling analysis of the used ceramic membranes. Sep. Sci. Technol..

[36] Carrère H., Blaszkow F., de Balmann H.R. (2001). Modelling the clarification of lactic acid fermentation broths by cross-flow microfiltration. J. Membr. Sci..

[37] Gao T., Wong Y., Ng C., Ho K. (2012). l-lactic acid production by *Bacillus subtilis* MUR1. Bioresour. Technol..

[38] Ma K., Hu G., Pan L., Wang Z., Zhou Y., Wang Y., Ruan Z., He M. (2016). Highly efficient production of optically pure l-lactic acid from corn stover hydrolysate by thermophilic *Bacillus coagulans*. Bioresour. Technol..

[39] Ye L., Zhou X., Hudari M.S.B., Li Z., Wu J.C. (2013). Highly efficient production of l-lactic acid from xylose by newly isolated Bacillus coagulans C106. Bioresour. Technol..

[40] Vincent-Vela M.C., Cuartas-Uribe B., Álvarez-Blanco S., Lora-García J. (2012). Analysis of an ultrafiltration model: Influence of operational conditions. Desalination.

[41] Hwang K.J., Wang C.Y. (2012). Microfiltration characteristics of Bacillus subtilis fermentation broths. J. Taiwan Inst. Chem. Eng..

[42] Ude C., Schmidt-Hager J., Findeis M., John G.T., Scheper T., Beutel S. (2014). Application of an online-biomass sensor in an optical multisensory platform prototype for growth monitoring of biotechnical relevant microorganism and cell lines in single-use shake flasks. Sensors.

[43] Giorno L., Chojnacka K., Donato L., Drioli E. (2002). Study of a Cell-Recycle Membrane Fermentor for the Production of Lactic Acid by Lactobacillus bulgaricus. Ind. Eng. Chem. Res..

[44] Fan R., Ebrahimi M., Quitmann H., Czermak P. (2016). Lactic acid production in a membrane bioreactor system with thermophilic *Bacillus coagulans:* Online monitoring and process control using an optical sensor. Sep. Sci. Technol..

[45] Suripto D.Y., Abdul G., Takao K. (2008). Effect of Product Inhibitions on l-Lactic Acid Fermentation from Fresh Cassava Roots in Tofu Liquid Waste by *Streptococcus bovis*. J. Ferment Bioeng..

[46] Zhang Y., Chen X., Luo J., Qi B., Wan Y. (2014). An efficient process for lactic acid production from wheat straw by a newly isolated *Bacillus coagulans* strain IPE22. Bioresour. Technol..

[47] Ahring B.K., Traverso J.J., Murali N., Srinivas K. (2016). Continuous fermentation of clarified corn stover hydrolysate for the production of lactic acid at high yield and productivity. Biochem. Eng. J..

[48] Yuwono S.D., Mulyono, Widiarto S., Hadi S., Kokugan T. (2014). Improvement of Lactic Acid Production from Cassava by *Streptococcus bovis* Using Two-Stages Membrane Bioreactor. Asian J. Chem..

[49] Van Hecke W., Verhoef S., Groot W., Sarić M., van de Bunt B., de Haan A., de Wever H. (2017). Investigation of lactate productivity in membrane bioreactors on C5/C6 carbohydrates. J. Membr. Sci..

[50] Tang J., Wang X.C., Hu Y., Ngo H.H., Li Y. (2017). Dynamic membrane-assisted fermentation of food wastes for enhancing lactic acid production. Bioresour. Technol..

[51] Tang J., Wang X., Hu Y., Zhang Y., Li Y. (2016). Lactic acid fermentation from food waste with indigenous microbiota: Effects of pH, temperature and high OLR. Waste Manag..

[52] Marques S., Matos C.T., Gírio F.M., Roseiro J.C., Santos J. (2017). Lactic acid production from recycled paper sludge: Process intensification by running fed-batch into a membrane-recycle bioreactor. Biochem. Eng. J..

[53] Mimitsuka T., Sawai K., Kobayashi K., Tsukada T., Takeuchi N., Yamada K., Ogino H., Yonehara T. (2015). Production of d-lactic acid in a continuous membrane integrated fermentation reactor by genetically modified Saccharomyces cerevisiae: Enhancement in d-lactic acid carbon yield. J. Biosci. Bioeng..

[54] Lee R.K., Ryu H.W., Oh H., Kim M., Wee Y.J. (2014). Cell-recycle continuous fermentation of *Enterococcus faecalis* RKY1 for economical production of lactic acid by reduction of yeast extract supplementation. J. Microbiol. Biotechnol..

[55] Min-tian G., Koide M., Gotou R., Takanashi H., Hirata M., Hano T. (2005). Development of a continuous electrodialysis fermentation system for production of lactic acid by *Lactobacillus rhamnosus*. Process. Biochem..

[56] Wang X., Wang Y., Zhang X., Xu T. (2012). In situ combination of fermentation and electrodialysis with bipolar membranes for the production of lactic acid: Operational compatibility and uniformity. Bioresour. Technol..

[57] Wang K., Li W., Fan Y., Xing W. (2013). Integrated Membrane Process for the Purification of Lactic Acid from a Fermentation Broth Neutralized with Sodium Hydroxide. Ind. Eng. Chem. Res..

